# *In vitro* and *in vivo* biological performance of porous Ti alloys prepared by powder metallurgy

**DOI:** 10.1371/journal.pone.0196169

**Published:** 2018-05-17

**Authors:** Renata Falchete do Prado, Gabriela Campos Esteves, Evelyn Luzia De Souza Santos, Daiane Acácia Griti Bueno, Carlos Alberto Alves Cairo, Luis Gustavo Oliveira De Vasconcellos, Renata Silveira Sagnori, Fernanda Bastos Pereira Tessarin, Felipe Eduardo Oliveira, Luciane Dias De Oliveira, Maria Fernanda Lima Villaça-Carvalho, Vinicius André Rodrigues Henriques, Yasmin Rodarte Carvalho, Luana Marotta Reis De Vasconcellos

**Affiliations:** 1 Department of Bioscience and Oral Diagnosis, Institute of Science and Technology, São Paulo State University (Unesp), São José dos Campos, São Paulo, Brazil; 2 Division of Materials, Air and Space Institute, Praça Mal. do Ar Eduardo Gomes, São José dos Campos, São Paulo, Brazil; 3 Department of Prosthodontic and Dental Material, Institute of Science and Technology São Paulo State University (Unesp), São José dos Campos, São Paulo, Brazil; 4 Department of Oral Diagnosis, Piracicaba Dental School, State University of Campinas (Unicamp), Piracicaba, São Paulo, Brazil; 5 Department of Restorative Dentistry, Institute of Science and Technology São Paulo State University (Unesp), São José dos Campos, São Paulo, Brazil; Universite de Technologie de Compiegne, FRANCE

## Abstract

Titanium (Ti) and Ti-6 Aluminium-4 Vanadium alloys are the most common materials in implants composition but β type alloys are promising biomaterials because they present better mechanical properties. Besides the composition of biomaterial, many factors influence the performance of the biomaterial. For example, porous surface may modify the functional cellular response and accelerate osseointegration. This paper presents *in vitro* and *in vivo* evaluations of powder metallurgy-processed porous samples composed by different titanium alloys and pure Ti, aiming to show their potential for biomedical applications. The porous surfaces samples were produced with different designs to *in vitro* and *in vivo* tests. Samples were characterized with scanning electron microscopy (SEM), energy dispersive spectroscopy (EDS) and elastic modulus analyses. Osteogenic cells from newborn rat calvaria were plated on discs of different materials: G1—commercially pure Ti group (CpTi); G2—Ti-6Al-4V alloy; G3—Ti-13 Niobium-13 Zirconium alloy; G4—Ti-35 Niobium alloy; G5—Ti-35 Niobium-7 Zirconium-5 Tantalum alloy. Cell adhesion and viability, total protein content, alkaline phosphatase activity, mineralization nodules and gene expression (alkaline phosphatase, Runx-2, osteocalcin and osteopontin) were assessed. After 2 and 4 weeks of implantation in rabbit tibia, bone ingrowth was analyzed using micro-computed tomography (μCT). EDS analysis confirmed the material production of each group. Metallographic and SEM analysis revealed interconnected pores, with mean pore size of 99,5μm and mean porosity of 42%, without significant difference among the groups (p>0.05). The elastic modulus values did not exhibit difference among the groups (p>0.05). Experimental alloys demonstrated better results than CpTi and Ti-6Al-4V, in gene expression and cytokines analysis, especially in early experimental periods. In conclusion, our data suggests that the experimental alloys can be used for biomedical application since they contributed to excellent cellular behavior and osseointegration besides presenting lower elastic modulus.

## Introduction

The use of implants for rehabilitation of lost body structures has shown remarkable advances, providing better quality of life for patients and longevity to implants [1–6]. Commercially pure Titanium (CpTi) and Titanium-6 Aluminium-4 Vanadium (Ti-6Al-4V) alloys are the most commonly used materials in surgical medical situations[7], due to their favorable mechanical properties, low cost, corrosion resistance and good tissue tolerance [8].

However, both have the disadvantage of the difference between the elastic modulus of the material and bone tissue. The metal implant produced from commercially pure Ti or Ti-6Al-4V alloy presents elastic modulus over 100 and 110GPa, respectively [1]. On the other hand, human cortical bone exhibits an approximate value of 20 GPa[1]. This difference may result in an inefficient transfer of the load from the implant to the adjacent bone (stress shielding). This phenomenon, observed as a bone resorption around the implant, may result in implant loss and possible bone fracture [3, 4, 9].

Ti-6Al-4V alloy has other disadvantages regarding the toxicity of its basic elements [10]. The development of new titanium alloys, which exhibit only biocompatible elements, aim to improve the properties of the material, such as corrosion resistance, biocompatibility, elastic modulus and ductility [3, 11, 12]. Binary or tertiary alloys containing the basic elements of niobium (Nb) and zirconium (Zr) have been investigated for their biocompatibility [3, 13, 14] and also because they present high corrosion resistance and lower elastic modulus than Ti-6Al-4V and Ti-6Al-7Nb alloys [7, 15]. Quaternary alloys such as Ti-Nb-Zr-Ta (titanium-niobium-zirconium-tantalum) are biocompatible [16] and have one of the lowest elastic modulus [17] besides exhibiting good mechanical resistance and an adequate limit in the fatigue test [18]. The combination of mechanical and biological aspects of Ti alloys make these metals desirable candidates for biomedical applications, mainly in the fields of orthopedics and dentistry [6, 19, 20].

The process of osseointegration with the new alloys seems to be favored [2, 6, 21, 22]. However, new methods to optimize osseointegration have been investigated, such as the alteration of the surface topography [2]. The formation of pores on the surface significantly increases the area of bone-implant contact due to bone growth in the interior of the pores (bone ingrowth), resulting in the acceleration of osseointegration [6, 23]. It also improves mechanical bonding and the interdigitation of bone tissue to the implant, resulting in long-term success [6, 19, 24].

Powder metallurgy (PM) is a promising technique that uses spacer particles to generate porous structures. The porosity of the materials depends on the amount of spacer particles used and it will determine the mechanical properties of the resulting material [25].

In dentistry, the use of porous surface implants is indicated, especially in patients exhibiting tiny height and low quality bone tissue [26–28] or even for orthodontic movements [29]. Additionally, due to the fact that porous implants promote greater contact between bone and implant surfaces, it may be used in both, critical defects and osseointegration in patients exhibiting changes in bone metabolism [28, 30, 31].

Biomaterials with porous surfaces have been even more attractive in implants if we take into consideration the great demand of patients requiring agility in restoring function, rehabilitative treatments in patients with low bone quality and/or with systemic disorders. Also, the increase in life expectancy request improves in the longevity of the devices in function. Thus, it is important to know the mechanisms by which the porous structure of CpTi or Ti alloys implants promote greater proliferation of the bone tissue. It is also important to evaluate the best material to be used for implants manufacturing, based on their biomechanical properties and surface characteristics.

In our previous studies, it has been shown that porous samples of Ti or binary alloys are adequate for osseointegration [11, 12]. We reported the development of a new design for Ti implants with porous surface integrated to dense core, in order minimize the difference of elastic modulus between metallic alloy and bone [20]. However, there is lack of research with porous β type Ti alloys produced by powder metallurgy. In this study, we aimed to assess the influence of different porous β type Ti alloys on osseointegration and osteogenesis by means of cellular, cytokines and molecular analyses *in vitro*, and histomorphometry and microcomputed tomography analysis *in vivo*.

## Materials and methods

### Samples: Preparation of experimental alloys

Samples were prepared with the powder metallurgy technique. The powders of the zirconium (purity 99.5%, <325 mesh), tantalum (purity 99.9%, <325 mesh) and niobium (purity 99.8%, <45 μm) were purchased (Sigma–Aldrich, St. Louis, MO, USA) and mixed to the powder of pure Ti grade II (CpTi) (purity 99.5%, < 8 μm), which was obtained by hydrogenation and dehydrogenation process (HDDH) at the Department of Science and Aerospace Technology (DCTA)—Materials Division of the Institute of Aeronautics and Space (AMR/IAE). Chemical composition of the experimental alloys powder is presented in Table 1.

**Table 1:** Chemical composition of the experimental alloys powder (wt %).

Alloy	Ti	Nb	Zr	Ta	O
Ti13Nb13Zr	Balance	13.1	13.2	-	0.19
Ti35Nb	Balance	35.3	-	-	0.19
Ti35Nb7Zr5Ta	Balance	35.3	6.99	5.4	0.19

**Fig 1:**
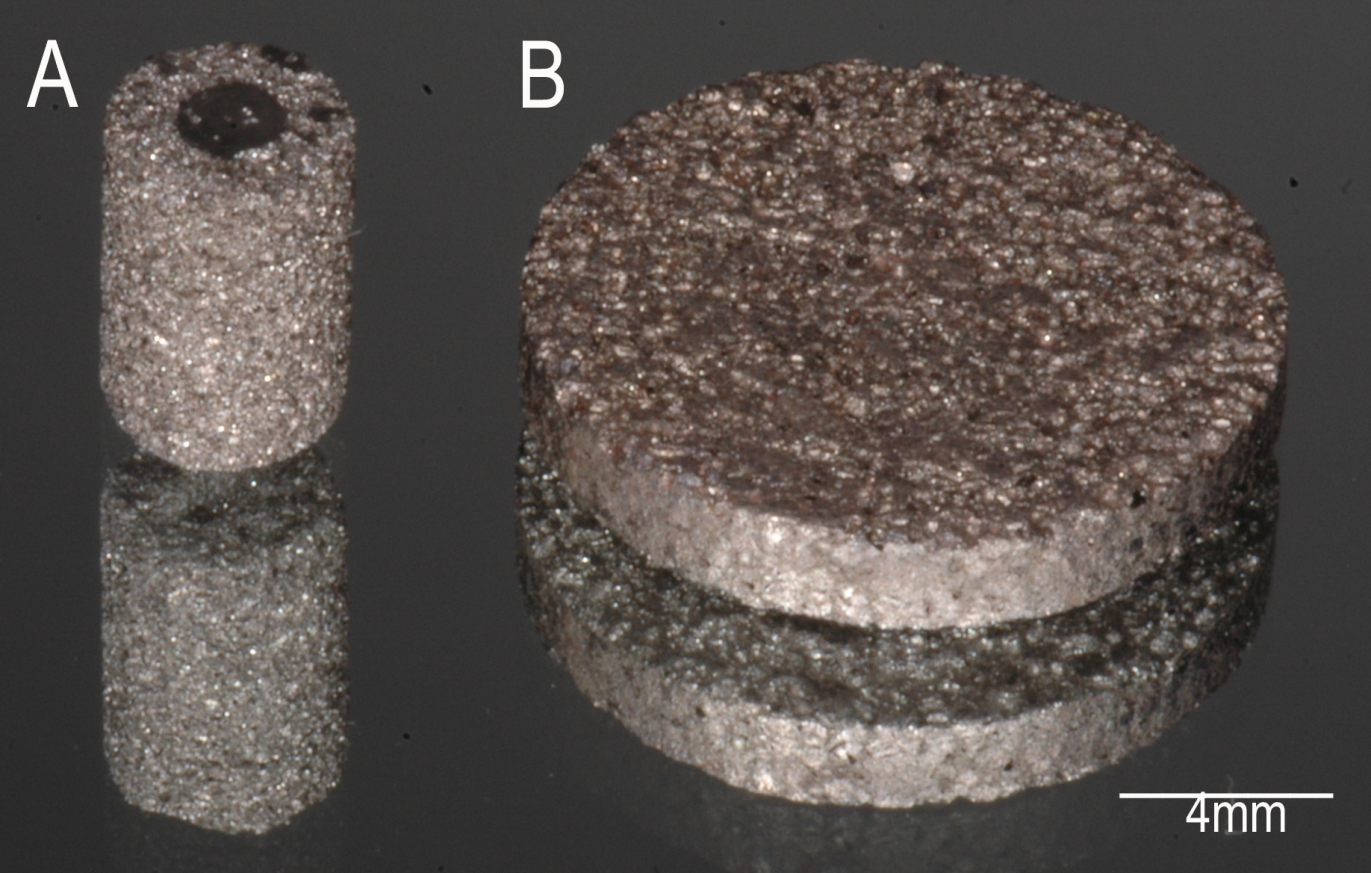
Macrostructure of samples. a) implant with porous surface integrated with a dense core; b) disc exhibiting a totally porous structure.

The alloy powders were mixed with urea (purity >98.0%, powder) (Sigma–Aldrich, St. Louis, MO, USA), an organic additive that functioned as a spacer, allowing the pores formation. The amount and particles size of urea used to obtain the mean diameter of the pores and the porosity percentage was based on previous studies performed by our research group[19, 20]. The powder metallurgy process is described in details in Vasconcellos et al., 2012[20]. Briefly, the powders of each alloy and urea were mixed in a planetary grinding machine to obtain a completely uniform mixture and compacted in two different designs: discs and cylindrical implants. Urea was removed and samples sintered. For *in vitro* study, the discs (Fig 1A) were manufactured according to Prado et al. 2015[12], exhibiting a totally porous structure and measuring 12 x 5 mm. For *in vivo* study, implant design with porous surface integrated with a dense core (Fig 1B), measuring 6 x 4 mm, were produced using the methodology described by Vasconcellos et al. 2012[20].

Additionally, CpTi powder (Sigma–Aldrich, St. Louis, MO, USA, purity 99.0%, 4 μm) was used, as positive control group, to produce discs and implants, in order to compare them with experimental alloys and Ti-6Al-4V alloy.

### Samples characterization

After production, five samples of each group were characterized by means of scanning electron microscopy (SEM) (Inspect S50—FEI—Montreal, QC, Canada) and energy dispersive spectroscopy (EDS) (Oxford, Carl Zeiss, Brazil), in order to analyze their microstructural features. Each sample was observed at three randomized selected areas.

Samples used for *in vivo* study were formed by dense core integrated to porous surface and this structure was also evaluated by SEM. The cylindrical samples were longitudinally sectioned and images were captured to observe the interface between dense core and porous surface.

The surface morphology was evaluated qualitative and quantitatively by means of metallography. Four images of each surface were captured and submitted to *Image J software* (Java 1.6.0_20/ 64-bit version), by means of *Straight* tool. The porous size and porosity were calculated by volumetric analysis of 5 samples of each group, using the relative density of each material, as described by Vasconcellos et al., 2008[19]. Additionally, to access geometry of pores in surface, *Angle* tool was used to perform the most acute angle measurement.

The elastic modulus was obtained using an universal testing machine (MTS 810), based in deformation x tension curve; performed using ASTM E09-09 standard. The values of elastic modulus were expressed in tension x deformation graphs, obtained in the compression test for each studied alloy. These values, expressed in GPa were obtained by means of linearization of the portion corresponding to the elastic zone of the graphs.

After procedures, the implants were properly washed in ultrasound and sterilized with 25kGy in Embrarad company (Sterilization Unit LTDA, Cotia- SP -Brazil).

### Biological assays: Isolation and culture of rat calvarial-derived cells

This research was approved by the Research Ethics Committee of the School of Dentistry of São José dos Campos/UNESP (018/2010/PA/CEP) and was carried out in accordance with the Ethical Principles for Animal Experimentation, adopted by the National Council of Control of Animal Experimentation (CONCEA). Cells from newborn (2–4 days) Wistar rat calvaria were harvested using the enzymatic digestion process previously described by Oliveira and Nanci[32]. Cells were seeded on samples in 24-well polystyrene plates and all *in vitro* tests were performed as described by Andrade et al., 2015[11]. Five samples from each group were used and all the assays were performed in triplicate (n = 12 well/ group), which were representative of three distinct primary cultures. All tests were developed in accordance with ISO-10993-5. A negative control group was performed and cells were plated in contact with the bottom of polystyrene culture plate wells.

### Cell viability

For the evaluation of cell viability, the cells were cultured on the samples at a concentration of 2 x 10^4^ viable cells/disc/well during 3 and 10 days. MTT assay was performed as described by Andrade et al., 2015[11]. Data was expressed as absorbance.

### Cellular differentiation

The total protein content (TPC) was determined using the same lysate of ALP, using modified Lowry method [33].

In order to determine alkaline phosphatase (ALP) activity, it was used the thymolphthalein monophosphate release assay, after 3, 10 and 14 days, with a commercial Kit (LabtestDiagnóstica, Belo Horizonte, BR), in accordance with the manufacturer’s recommendations. Absorbance was measured at a wavelength of 590 nm in a UV 1203 spectrophotometer (8582 Micronal, São Paulo, Brazil). The results were expressed in μmol of thymolphthalein/min/mL.

After 14 days in culture, the mineralized bone-like nodule formation was accessed using 2% Alizarin red S (Sigma) staining, pH 4.2, for 15 min at 37°C. The quantification was performed according to the method previously described by Gregory et al.[34]; at a wavelength of 405 nm in a spectrophotometer. The values were expressed in optical density.

### Cytokines expression

After 7 and 14 days, the production of TGF-β1, TNF-α and IL-6was evaluated by ELISA (DuoSet1 ELISA kits for rat—R&D Systems, Minneapolis, MN, USA), according to the recommendations of the manufacturers. Briefly, the plates were coated with capture antibody and kept overnight. The next day, the samples and standard curve were added and after incubation, the microplates were incubated with biotinylated detection antibody in the wells. The reaction was performed with a chromogen solution. The absorbance was measured with theid of a plate reader at a wavelength of 450 nm and the results were converted into concentrations (pg/mL), using GraphPad Prism 5 software.

### Gene expression of osteogenic markers

Gene expression of alkaline phosphatase (ALP), osteocalcin (OC), osteopontin (OP) and runt-related transcription factor 2 (Runx_2_) was evaluated after 7 days of culture with the constitutive gene GAPDH by means of relative quantification by reverse-transcriptase PCR in real time. All the methodology used was previously described [12]. RT-PCR was performed with detection system Step one Plus 7000 Real Time PCR Systems (Applied Biosystems), using Platinum SYBR Green qPCR Super Mix-UDG (Invitrogen Life Technologies Corporation-Van Allen Way, Carlsbad, California, USA) and specific primers (Table 2). The RT-PCR reactions were performed in triplicate.

**Table 2:** Description of primers with sense and antisense sequence, melting temperatures, product size in base pairs and PUBMED reference.

Gene	Primers	Product Length (BP)	FASTAPUBMED Reference
GAPDH	GGCACAGTCAAGGCTGAGAATG	143	NM_017008.4
	ATGGTGGTGAAGACGCCAGTA		
Alkaline phosphatase	TATGTCTGGAACCGCACTGAAC	192	XM_006239136.3
	CACTAGCAAGAAGAAGCCTTTGGG		
Osteocalcin	GAGGGCAGTAAGGTGGTGAA	154	XM_006232594.2
	CGTCCTGGAAGCCAATGTG		
Osteopontin	ATCTGATGAGTCCTTCACTG	151	NM_012881.2
	GGGATACTGTTCATCAGAAA		
Runx2	GCCGGGAATGATGAGAACTA	200	NM_001278483.1
	GGACCGTCCACTGTCACTTT		

### Surgical procedure for in vivo analysis

Ten New Zeland rabbits, weighing approximately 4.0 kg, 5 months old, were used in this study. The animals were placed in individual cages and received standard commercial food and distilled water ad libitum. The protocol was also approved by the Research Ethics Committee of the School of Dentistry of São José dos Campos/UNESP (018/2010/PA/CEP).Previously to procedures, the rabbits were weighed and anesthetized with intramuscular injection of aqueous solution (13 mg/kg of 2% hydrochloride of 2-(2,6-xylidine)-5,6-dihydro-4H-1,3-thiazine (Rompun®, Bayer, São Paulo, Brazil), analgesic, sedative and muscular relaxant, and 33 mg/kg of ketamine (Dopalen®, Agibrands do Brazil Ltda., São Paulo, Brazil), as general anesthetic, as described in our previous study[20]. Implants of CpTi, Ti-6Al-4V and Ti-13Nb-13Zr, were inserted in the right tibia, starting from the medial to the distal portion. In the left tibia, samples of Ti-35Nb, Ti-35Nb-7Zr-5Ta were inserted. In the left tibia, samples from Groups 4 and 5 were inserted. The implants were placed under pressure until they were attached to the cortical bone, opposite the perforation.

Next, all of the rabbits received one dose of antibiotic, 0.35 mg/kg (Pentabiotico®, Fort Dodge Saúde Animal, São Paulo, Brazil). The animals were inspected daily for clinical signs of complications or adverse reactions, in order to prevent any suffer to animals. They were monitored until the euthanasia period, between 2 and 8 weeks (n = 5). After euthanasia, all bone fragments containing the implants were fixed in 10% neutral-buffered formalin solution for microcomputed tomography (μCT) and histomorphometric analyses.

### Microcomputed tomography

After euthanasia, the bone segments were kept in 10% formalin buffered, pH 7.0, for 48 h and then transferred to a solution of 70% ethanol for 72 h. Then, the samples were submitted the three-dimensional (3D) microcomputed tomography analyses using SkyScan 1172 (Kontich, Belgium). Tomographic images were acquired at 70 keV and 702 μA, 500 projections/180° rotation and 300 ms integration and images were reconstructed using the software NRecon (Bruker-Skyscan). The analyses of three-dimensional volume of interest (VOI) were determined with a radius of 1 mm from implant surface. Thresholding was performed in Mode Global; Lower grey threshold, 25; Upper grey threshold, 65. Then, after running “3D analysis” data was obtained according the principal bone structural parameters: bone volume (BV), trabecular number (Tb.N), total volume (TV) used to calculate the bone volume fraction (BV/TV). These parameters were automatically determined from the region of interest using the 3D CT Analyser (V1.11.4.2, Skyscan 1172, Kontich, Belgium).

### Histological and histomorphometric analysis

After microcomputed tomography analyses, bone segments containing the implants were dehydrated in a graded alcohol series and embedded in methylmetacrylate. Three nondecalcified sections were obtained using a diamond saw in a cutting machine for hard tissues (Labcut 1010, Extec, USA) as previously described by Vasconcellos et al. (2008)[19]. Initially, the histological description of interface tissues bone-implant were observed on light microscope (Axioplan 2, Carls Zeiss, Germany). Next, the histomorphometric analysis was performed using an association of microscopy with a Sony digital camera (DSC-S85, Cyber-shot). The bone ingrowth was evaluated by a calibrated blinded investigator, using 2 different images of both sides of each section of the bone-implant contact (BIC), since three sections were obtained from each implant on each of the five different rabbits. BIC area analyzed was previously determined as the pores in contact to preexistent cortical bone in original magnification 10X. Bone ingrowth into the interior of the pores was calculated using Image J software, version 1.34 (NIH).

### Statistical analysis

After testing the normal distribution (Kolmogorov-Smirnov test), differences were analyzed by statistical tests. Data are represented by mean and standard deviation graphs. Data were analyzed using GraphPad Prism 5.0 software. The analysis of variance ANOVA one factor was performed to cell viability, ALP, PTC, cytokine expression tests. One Way ANOVA was performed to polymerase chain reaction in real time RT-PCR, μCT and quantification of mineralized nodules data. In case of significant differences, Tukey test with significance level of 5% was used. Metallographic data was analyzed with Kruskall Wallis test (p<0.05).

## Results

### Sample characterization: Metallographic and EDS analyses

All material formed a porous structure after sintering by means of powder metallurgy technique. The surface exhibited interconnected macro and micropores. Some isolated pores were observed as result of volume shrinkage from the sintering process of the titanium powders. The larger interconnected pores were successfully fabricated as result of the elimination of the organic additive. These pores are important to the ingrowth of new bone tissue and the transportation of body fluids. All the samples had similar pore sizes, with the pore size distribution within each sample ranging approximately from 64 to 200 μm, with a mean diameter of 99,5 μm. Statistical analysis (with Kruskal-Wallis test; p = 0.0521) showed no significant differences among groups (TiCp = 113μm (±24.44); Ti-6Al-4V = 104.1 μm (±16.16); Ti-13Nb-13Zr = 105 μm(±23.37); Ti-35Nb = 72.87 μm (±8.53) Ti-35Nb-7Zr-5Ta = 102.7 μm (±15.68). Porosity varied between 39.06% (±1.09%) (CpTi) and 47.89% (±4.53%) (Ti-35Nb), with a mean of 42%. Statistical analysis (with Kruskal-Wallis test; p = 0.104) revealed no significant differences among groups; Ti-6Al-4V = 40.61% (±7.81%); Ti-13Nb-13Zr = 39.91 (±6.35%) and Ti-35Nb-7Zr-5Ta = 42.55% (±7.8%).

Statistical analysis of geometrical characteristic of pores showed no significant differences among groups with ANOVA test; p = 0.8641. TiCp presented mean of 73.45°(±26.32°); Ti-6Al-4V = 85.65 °±16.53°; Ti-13Nb-13Zr = 75.68°±27.44°; Ti-35Nb = 70.55°±17.58°and Ti-35Nb-7Zr-5Ta = 77.10°± 32.19°.

**Fig 2:**
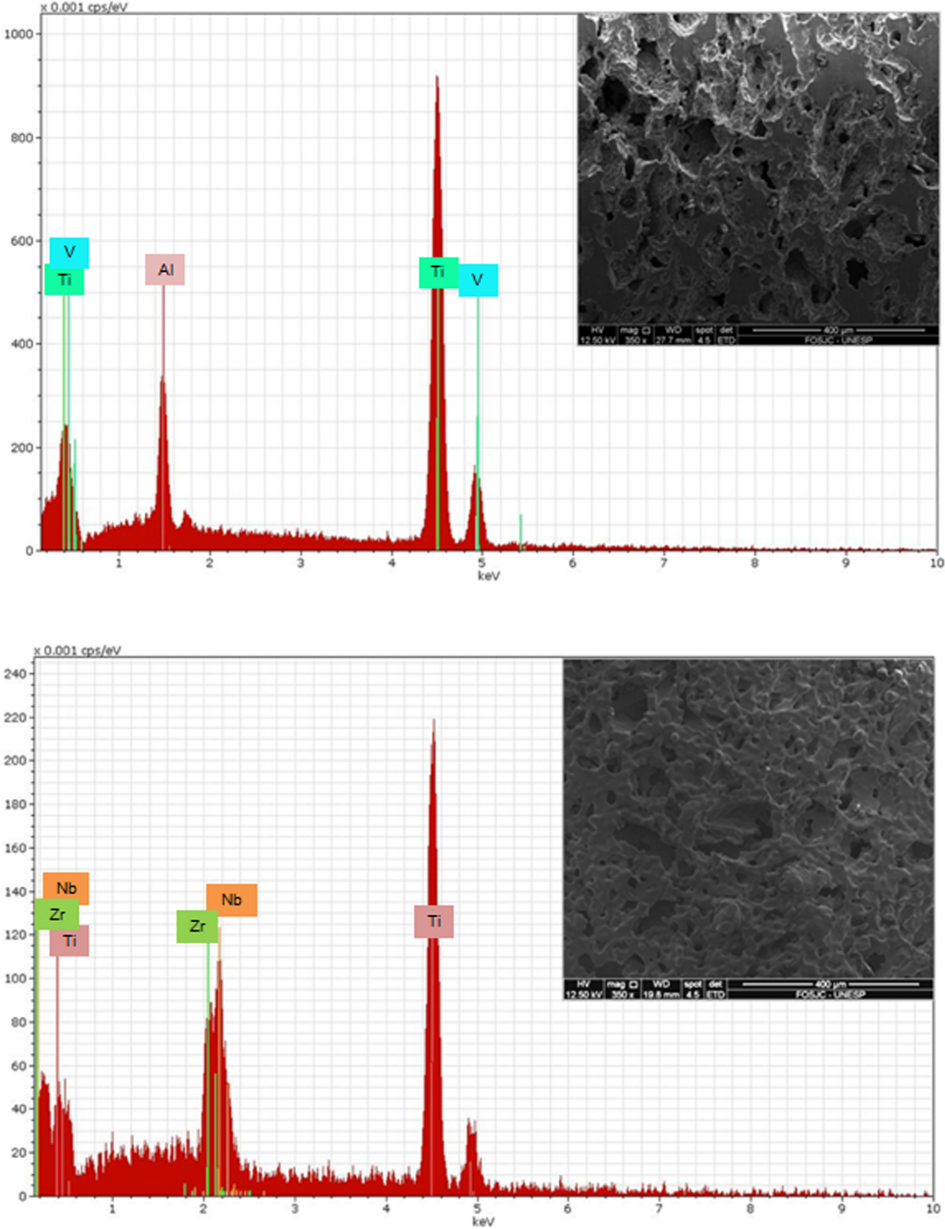
EDS results and SEM image. a) Ti-6Al-4V alloy; b) Ti-13Nb-13Zr alloy.

**Fig 3:**
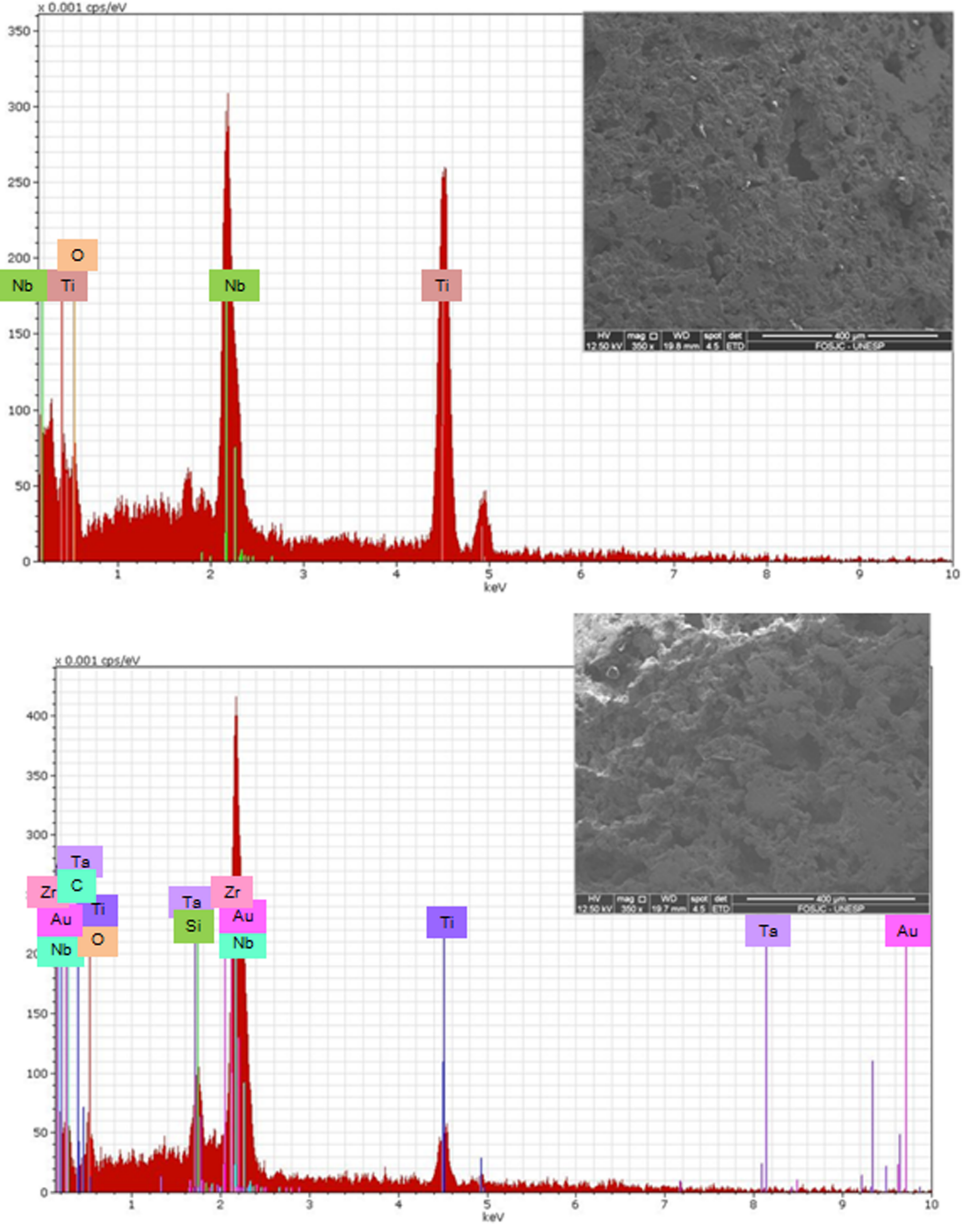
EDS results and SEM image. a) Ti-35Nb alloy; b) Ti-13Nb-13Zr alloy.

Results of EDS (Figs 2 and 3) show the main elements presents on the samples.

**Fig 4:**
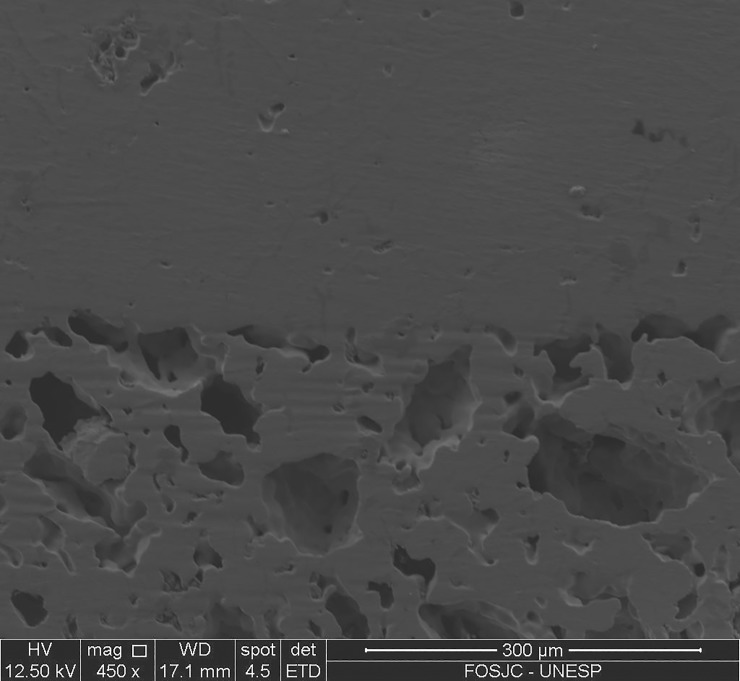
Scanning electron micrograph of a longitudinal section. a) implant with porous surface integrated with a dense core; b) detail of pores interconnected.

As previously demonstrated by our research group[20], the implants with porous surface integrated with a dense core do not exhibit fracture line between the surface and core (Fig 4).

**Fig 5:**
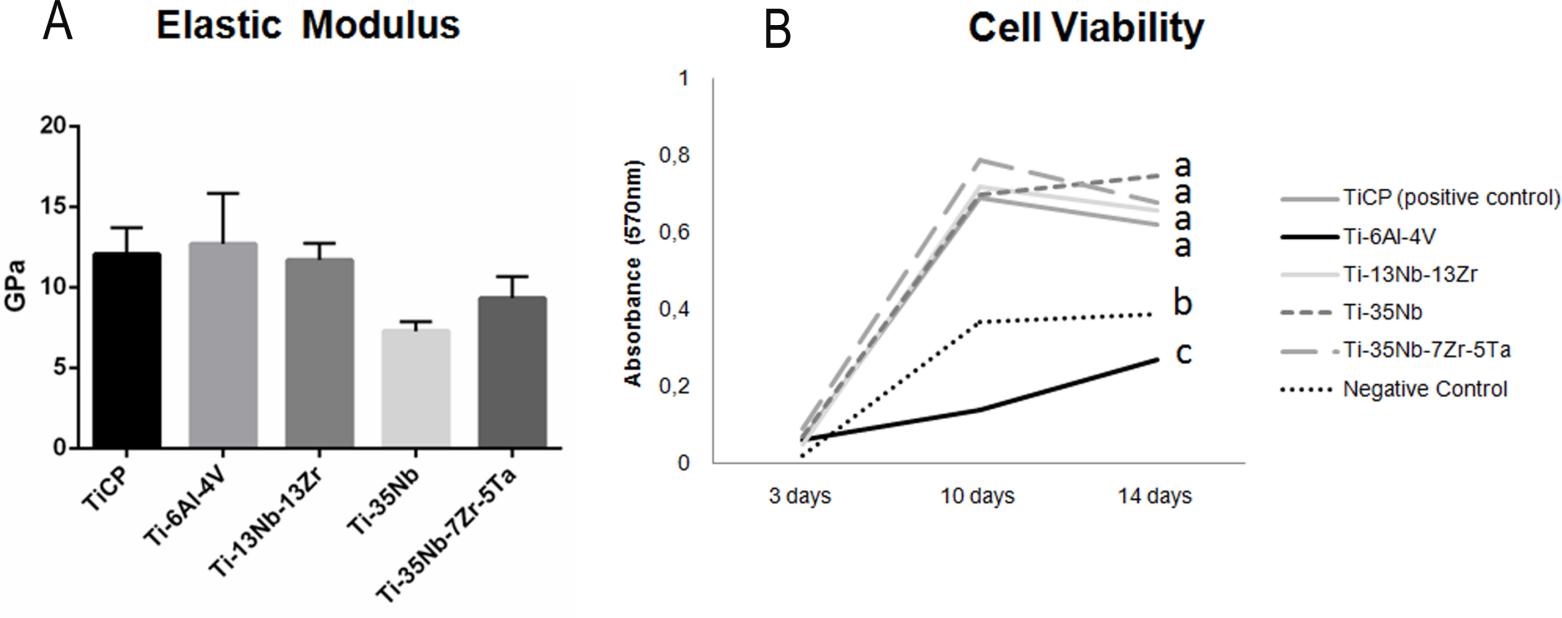
Graphics of elastic modulus and effects of sample and periods in cell viability. A- Mean and standard deviation of elastic modulus of each group B- Results of MTT assay. Tukey tests results are presented in superscript letters.Values that do not share the same superscript letters are significantly different from each other (P < 0.05).

Descriptive statistics revealed that Ti-6Al-4V alloy showed the highest elastic modulus (12,87GPa), while Ti-35Nb experimental alloy showed the lowest mean (7,7GPa) (see Fig 5A). CpTi, Ti-13Nb-13Zr and Ti-35Nb-7Zr-5Ta alloys exhibited intermediary values: 12,01GPa, 11,76GPa and 9,21GPa, respectively. However, no significant difference was observed among the groups (p>0.05).

### In vitro analysis: Cell viability

Cell viability analyses indicated that cell number increased from days 3 to 10, independently of the material (p<0.05), and generally decreased at 14 days, excepted for Ti-6Al-4V and Ti-35Nb alloys (Fig 5B). It was not observed difference between 10 and 14 days (p>0.05). Finally, the lowest number of viable cell was observed in the Ti-6Al-4V alloy, differing from all groups (p<0.05). The negative control group (polyestyrene well) also showed low cell viability, differing from other groups (p<0.05). The highest number of viable cell was observed in the Ti-35Nb-7Zr-5Ta sample but without significant difference with CpTi, Ti-35Nb and Ti-13Nb-13Zr alloys (p<0.05).

### Total protein content (TPC)

**Fig 6:**
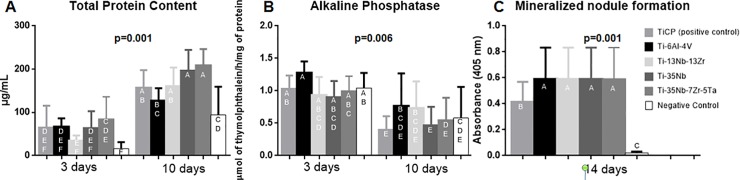
Graphics of data showed effect of sample and periods in different analyses. a) total protein contend; b) ALP; c) mineralized matrix.

TPC data were analyzed by Two-way ANOVA test, which demonstrated significant difference for all the factors (p<0.05): type of alloy, time of culture and interaction between them (Fig 6A). The highest TPC was observed in the Ti-35Nb-7Zr-5Ta alloy, differing from Ti-6Al-4V and Negative Control group at 10 days and from all groups at 3 days (p<0.05). At 3 days, the negative control group showed the lowest TPC, differing from the Ti-35Nb-7Zr-5Ta alloy (p<0.05) and from all groups at 10 days (p<0.05).

### Alkaline phosphatase activity

The results of ANOVA test showed that there was significant difference according to the type of alloy (p<0.05) and periods of cell culture (p<0.05) but there was no difference in the interaction of factors (p>0.05). ALP activity decreased between 3 and 10 days (p<0.05) for all samples, but no difference among the groups was observed in this period (p<0.05) (Fig 6B).

### Mineralized bone-like nodule formation

Bone-like nodule formation at 14 days was influenced by the type of alloy (p<0.05). Ti-35Nb-7Zr-5Ta and Ti-35Nb groups demonstrated the highest amounts of nodules. Significant difference was observed among these groups and the other groups. The lowest formation of nodules was observed in Ti-13Nb-13Zr alloy, differing from the other groups. (Fig 6C).

### Cytokine production

Regarding TNF-α production (Table 3), two way ANOVA test demonstrated that the type of alloy (p = 0.002) was statistically significant, while culture time (p = 0.915) and the interaction between the two factors (p = 0.946) did not provide significant difference. At 7 days, there was difference only between negative control group (0.199 pg/mL) and Ti-35Nb-7Zr-5Ta (92,527 pg/mL).

**Table 3:** *In vitro* production of proinflammatory cytokines by cells in response to each alloy.

	*TiCP*	*Ti-6Al-4V*	*Ti-13Nb-13Zr*	*Ti-35Nb*	*Ti-35Nb-7Zr-5Ta*	*Negative Control*
***IL6–7 days***	498,2±693,1^D^	103,9±99,97^D^	451,7±582,1^D^	918,5±823,5^CD^	425,8±368,6^D^	758,6±945,6^D^
***IL6–14 days***	4863±2435^AB^	3184±2985^BC^	5340±2293^AB^	5705±1903^A^	5489±2533^AB^	574,3±633,5^D^
***TNF-α - 7 days***	22,07±41,54	49,84±83,53	58,02±84,31	84,27±113,4	101,7±106,6	0,0±0,0
***TNF-α - 14 days***	30,26±49,64	75,37±113,9	66,43±106,1	69,07±80,61	83,32±94,34	0,3986±1,381
***TGF-β - 7 days***	781,4±96,75^C^	696,4±145,5^C^	762,5±82,94^C^	763,6±137,5^C^	779,8±59,90^C^	792,6±25,28^C^
***TGF-β - 14 days***	1259±142,4^AB^	1054±166,8^B^	1136±222,5^B^	1264±139,3^AB^	1438±145,8^A^	1378±197,2^A^

Mean (±standard deviation) of cytokines (pg/mL). Statistically significant difference among experimental groups can be observed with different superscript letters (A;B;C;D) in the table. (n = 8, ANOVA, Tukey Test, p≤0,05)

Statistical analysis for IL-6 release (Table 3) showed that its expression increased at 14 days for all samples (p<0.05). Ti-6Al-4V alloy showed the lowest mean at 14 days, while Ti-35Nb had the highest mean in this period, with significant difference (p<0.05). ANOVA two factors showed that the alloys, culture time and the interaction between the two events showed significant difference (p = 0.001).

In the two way ANOVA statistical test, it was observed that the type of alloy of the samples (p<0.05), culture period (p<0.05) and interaction between them presented significant difference (p<0.05) when TGF-β1 production was evaluated. At 7 days, there was no difference among the materials (p>0.05). On the other hand, at 14 days, Ti-35Nb-7Zr-5Ta exhibited higher TGF-β1 production than Ti-13Nb-13Zr and Ti-6Al-4V alloys, which showed the lowest values of TGF-β1 (p<0.05). TGF-β1 production increased over the time in all the materials, resulting in the highest production at 14 days (Table 3).

### Gene expression of osteogenic markers

**Fig 7:**
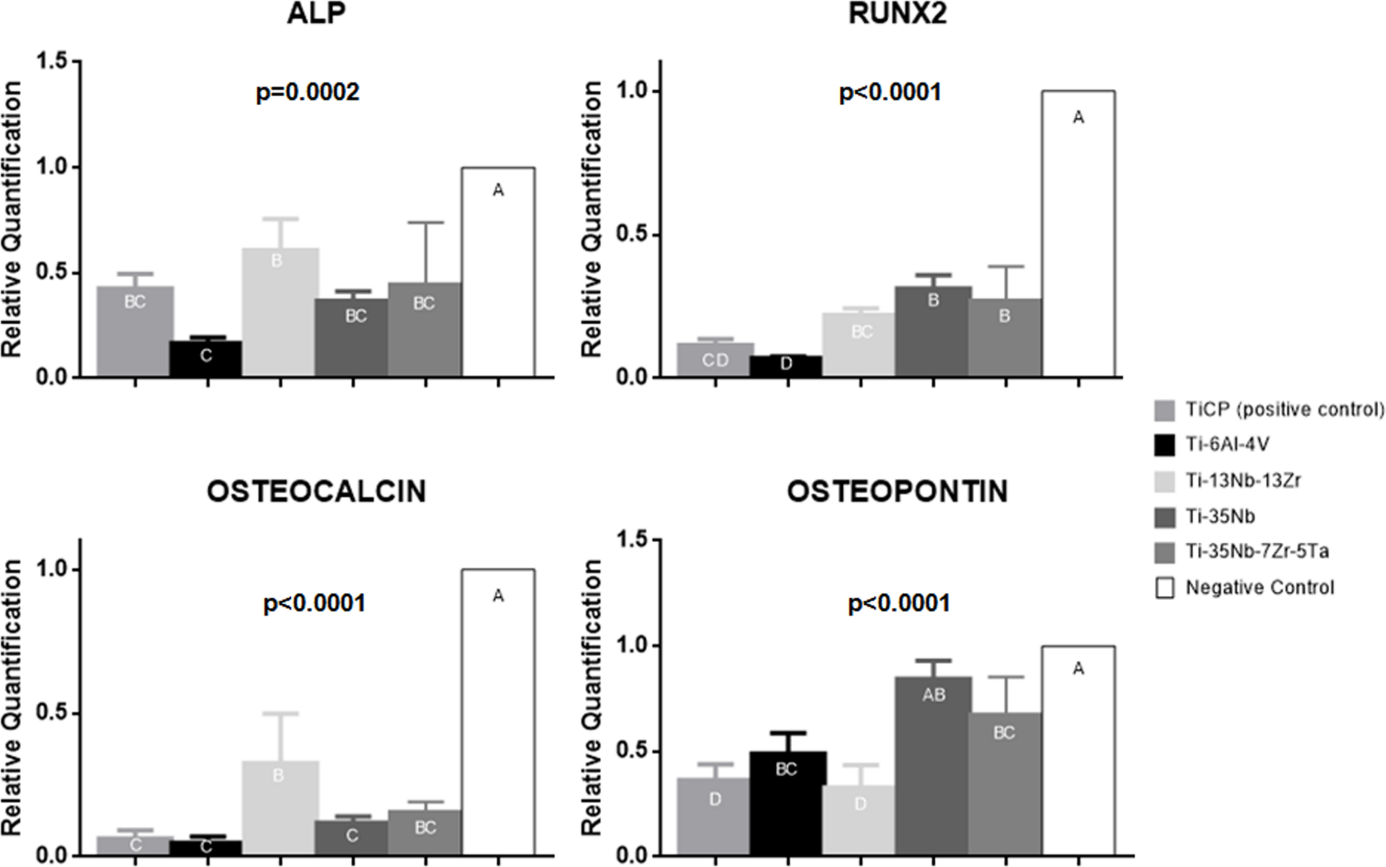
Data of molecular analysis, relative quantification of genes, after 7 days in contact with each group. a) alkaline phosphatase (ALP), b) Runx-2, c) osteocalcin, d) osteopontin. Different letters represent significant difference among the groups.

All data of real time qPCR can be found in Fig 7. At 7 days, Ti-13Nb-13Zr alloy showed the best result for ALP expression but it differed only from Ti-6Al-4V, that showed the lowest value for ALP (p>0.05). In this period, the negative control group exhibited the highest value when compared to the other materials (p<0.05).

The three experimental alloys showed similar values of Runx-2 gene among each other and, when compared to Ti-6Al-4V and CpTi alloys, significant difference was found (p<0.05), except for the comparison between CpTi and Ti-13Nb-13Zr alloys. Once again, negative control group showed the highest Runx-2 expression, differing from all the other groups (p<0.05).

Regarding OCN at 7 days, Ti-13Nb-13Zr alloy showed the highest its expression, being statistically different from CpTi, Ti-6Al-4V and Ti-35Nb alloys (p<0.05). However, Ti-13Nb-13Zr alloy did not differ from Ti-35Nb-7Zr-5Ta alloy. Negative control group differed from all materials, showing the highest expression of OCN (p<0.05).

Ti-35Nb alloy showed highest OPN expression at 7 days, being similar to Ti-6Al-4V, Ti-35Nb-7Zr-5Ta alloys and negative control group (p>0.05), which exhibited highest value of OPN and statistically differed from these alloys (p<0.05). However Ti-35Nb showed significant difference when compared to CpTi and Ti-13Nb-13Zr alloys, that presented the lowest values, and also differed from all others groups (p<0.05).

### Microcomputed tomography

**Fig 8:**
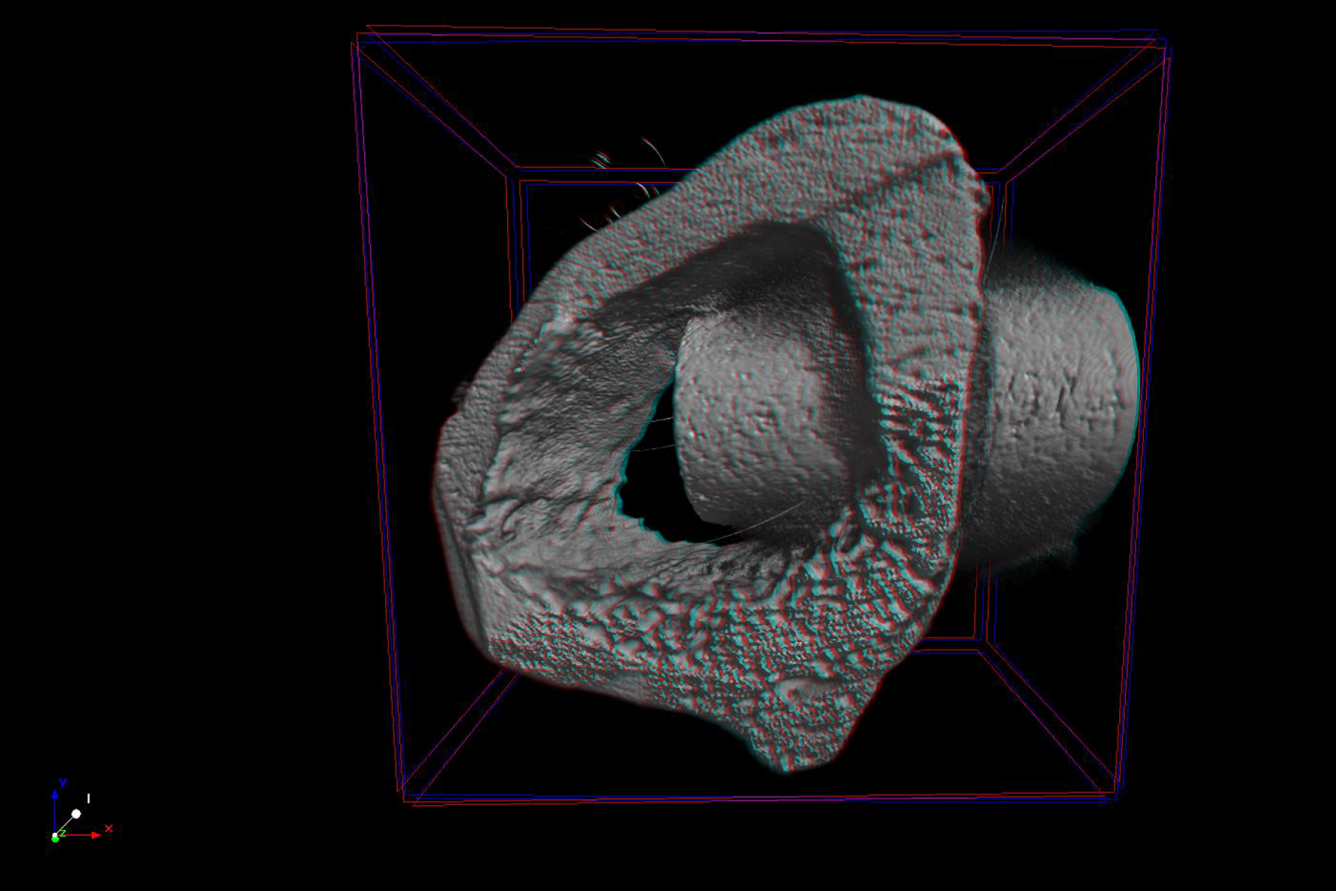
3D representation of micro-computed tomography. Image of implant and bone around implant at 4 weeks.

Reconstructed micro-CT images of longitudinal sections along the central axis of dental implants (Fig 8) proved that neoformed bone has expanded and occupied pores region.

**Fig 9:**
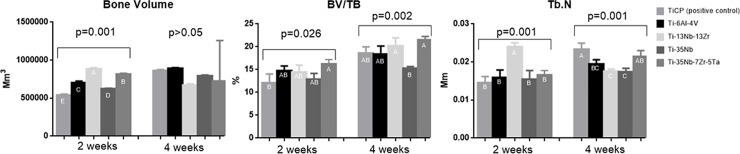
Graphics of values obtained by 3-dimensional reconstructed micro-computed tomography after 2 and 4 weeks of osseointegration in rabbit tibia. a) Bone volume (BV); b) bone volume fraction (BV/TV, c) trabecular number (Tb.N). Data are presented as mean ± standard deviation. The inverted bracket determines the groups compared in ANOVA. Significant P values are presented and different letters of Tukey test represent statistical significant among the groups.

Based on the results of bone volume (BV), at 2 weeks, all groups exhibited statistically difference (p<0.05), demonstrating that the material influenced the early bone proliferation. The highest BV was found in Ti-13Nb-13Zr alloy while the lower value was observed in CpTi (Fig 9). At 8 weeks, there was no significant difference among all the groups (p>0.05).

Regarding BV/TV proportion, at 2 weeks, Ti-35Nb-7Zr-5Ta alloy presented the highest proportion, but statistically differed only from CpTi (p<0.05), which exhibited the lowest proportion. At 8 weeks, Ti-35Nb-7Zr-5Ta alloy maintained the highest proportion but, this time, statistically differing only from Ti-35Nb alloy (p<0.05).

Regarding Tb.N, at 2 weeks, Ti-13Nb-13Zr alloy showed higher number, statitistically different from all the other groups (p<0.05). At 8 weeks, CpTi showed the highest Tb.N, statistically similar only to Ti-35Nb-7Zr-5Ta alloy (p>0.05). The lowest values were exhibited by Ti-35Nb and Ti-13Nb-13Zr alloys (Fig 9), which differed from the groups with higher values (p<0.05) but there was no difference with Ti-6Al-4V (p>0.05).

### Histological and histomorphometric analysis

**Fig 10:**
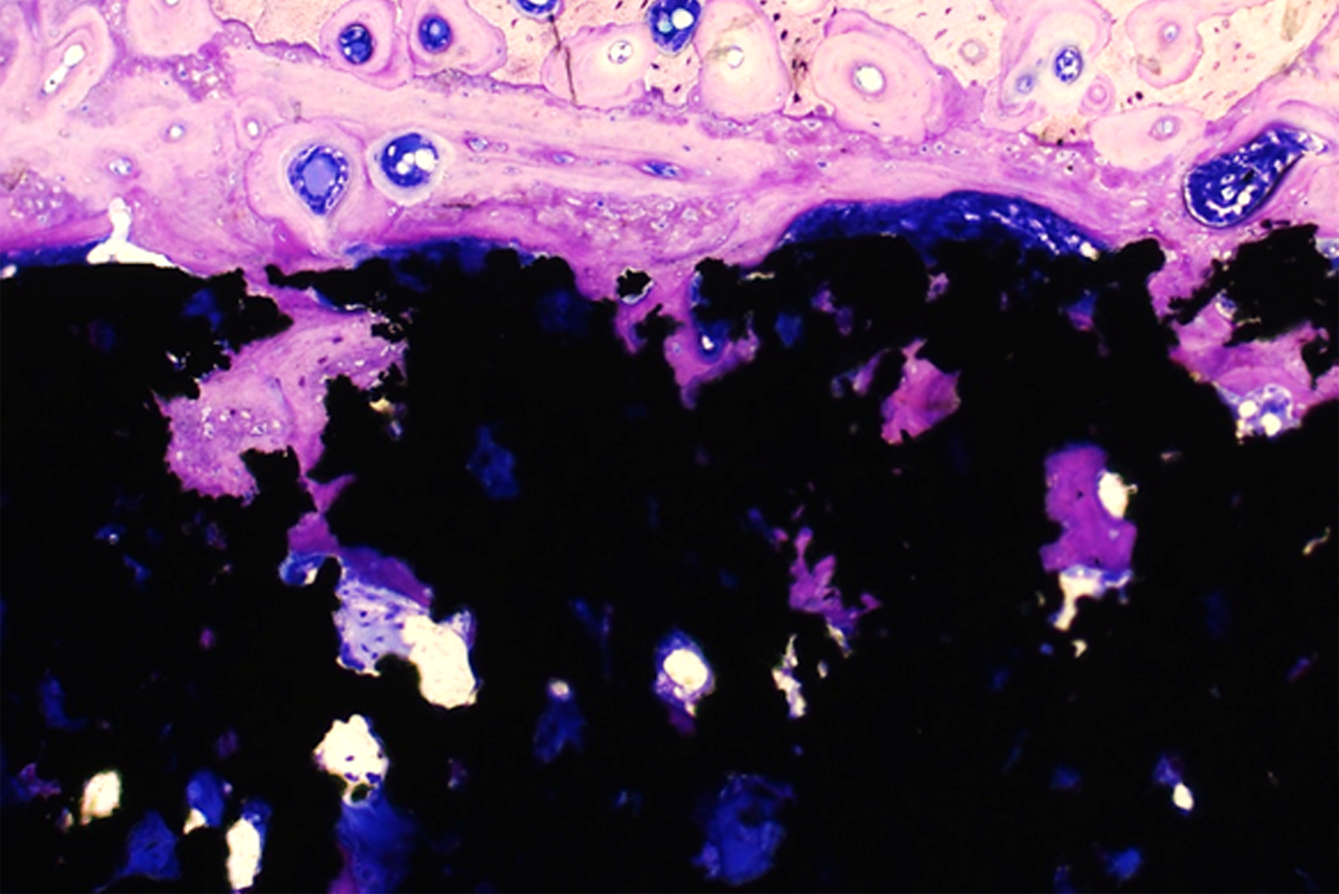
Photomicrography stained in toluidin blue. Osseointegration after 8 weeks of implantation demonstrating mature bone fused to cortical bone and the process of bone ingrowth to the pores.

In the histological evaluation, it was not observed inflammatory response in either group. The bone architecture was similar in both experimental periods (2 and 8 weeks), presenting osteoblasts and osteocytes in the new bone trabeculae. Specially at 2 weeks, osteoid material and many reversal lines were observed. An important histological difference was demonstrated in osseointegration at 8 weeks, when much more deeper pores were filled (Fig 10).

**Fig 11:**
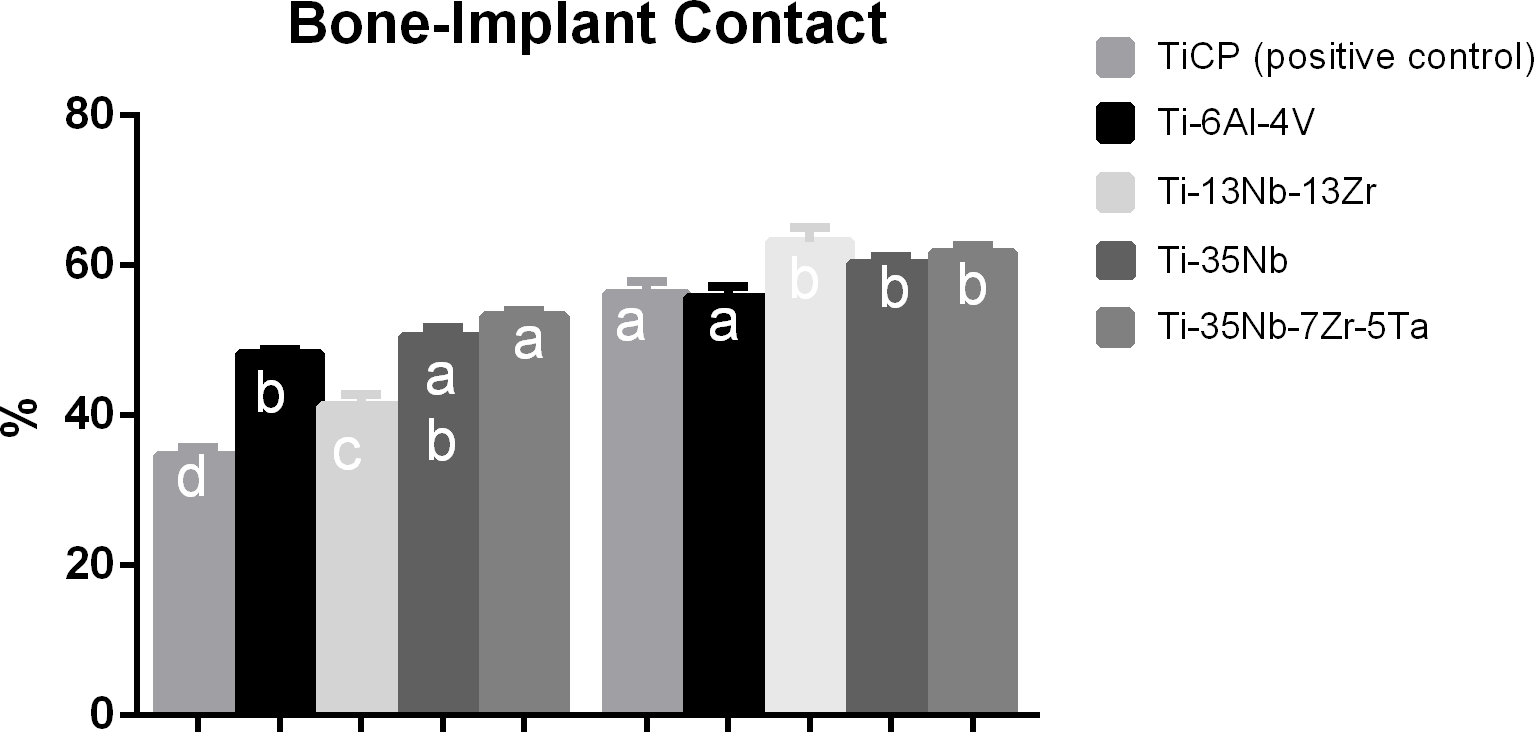
Graphic of values obtained by bone implant contact analysis. Data expressed in percentage. *Different letters represent significant difference among the groups.

At 2 weeks, the highest percentages of BIC were observed in Ti35-Nb-7zr-5Ta and Ti-35Nb implants. On the other hand, the lowest percentage of BIC was observed in CpTi, which differed from all groups (p<0.05). At 8 weeks, the experimental alloys exhibited the highest values of BIC, which statistically differed from CpTi and Ti-6Al-4V (p<0.05) (Fig 11).

## Discussion

About 70 to 80% of biomedical implants are made of metallic materials, and this demand has increased over the years, due to the increase in life expectancy of the population. Even with these increasing numbers, biological and mechanical properties of metallic biomaterials are not ideal, requiring improvements [3]. In this sense, the development of new materials presenting a chemical composition that stimulates favorable responses in the organism have been the aim of researches in the field of biomedical implants and biomaterials. Many of them, also aim to design the dental implant with a morphology that facilitates and reduces the time of osseointegration[20]. It has been shown that the architecture of the porous biomaterials may provide a favorable environment for cell growth[35]. We compared the *in vitro* and *in vivo* biological performance of experimental alloys to CpTi and Ti-6Al-4V. The results showed that experimental alloys promote the culture growth and, generally, enhance the matrix mineralization production as indicated by *in vitro* tests and induce early acquisition of the osteoblastic phenotype as demonstrated by gene expression profile, and supports the bone formation in close contact with porous implant specially in short time.

In the last decades, new alloys have been developed to solve the problems of elastic modulus, alloy-bone tissue incompatibility and to accelerate the osseointegration process [3, 5, 6, 15, 36]. The incorporation of new elements to Ti, such as Nb and Ta aims the maintenance and predominance of β phase in Ti, since β-type alloys show lower elastic modulus (below 85 GPa)[15].

All the experimental alloys evaluated in this study, exhibited a lower elastic modulus when compared to CpTi and Ti-6Al-4V alloy, but the values were not statistically different. We suggest that this result was obtained due to the powder metallurgy technique. After sintering there were residual pores that interfered in the values of the elastic modulus.

However, it is remarkable that this technique proved to be effective for the production of the idealized samples, and did not interfere in the chemical composition of each group, as observed by EDS. The powder metallurgy allowed the fabrication of an interconnected-pore structure surface, with satisfactory size, integrated with a dense core. Porous implants designs are investigated because they may improve bone ingrowth into surfaces and reduce the difference between bone and metallic surface elastic modulus, besides preventing aseptic loosening of implants and increasing their long-term stability [6]. The percentage and diameter of the pores promote a positive effect on the behavior of osteoblasts [19, 24, 37] since it mimics bone marrow tissue[38]. However, the size of the pores is of great importance; it must allow the formation of a new vascular system for the continuity of bone growth towards the interior of the pores[19, 37]. In the present study, the pore mean size of 99.53 μm and porosity around 45% were in agreement with previous studies [3, 20] and proved to be suitable for cell proliferation and differentiation, as verified by means of *in vitro* tests and by the osseointegration of the implants.

The different materials, tested in the present study, promoted different cellular responses and, in general, the experimental alloys exhibited the best results. In most cases, significant difference was observed between experimental alloys and CpTi or Ti-6Al-4V alloy, results that agree with previous studies [3, 39, 40]. Experimental alloys presented higher results, in cell viability and total protein and statistically differed from Ti-6Al-4V alloy at 10 days, as previously reported [8, 31, 40]. The lowest cell viability, observed in Ti-6Al-4V alloy, in different cell lines, may be the result of vanadium and aluminum ions release [39].

During the osteoblasts differentiation, the expression of Runx-2, followed by activation of alkaline phosphatase are very important in the process of bone formation. Runx2 is one of the main markers of initial differentiation of the osteoblasts [41], and in the present study, the experimental alloys showed higher expression of this marker, differing from Ti-6Al-4V alloy, in agreement with the previous study performed by Sista et al. (2013)[8]. Regarding ALP, it can be analyzed by methods of quantification at cellular [4, 11, 40] or molecular level [8, 12, 22]. In general, all the experimental alloys in this research induced consistent alkaline phosphatase data, both in cell and molecular analyses. Their values, from RT-PCR, were similar or higher than CpTi and Ti-6Al-4V samples, which are widely used in the medical-dental market. The groups did not demonstrate differences in ALP activity on cell culture analyzes, in agreement with the results obtained by Shi et al., (2013)[31] and Samuel et al., (2010)[40]. However, Guo et al. (2013)[4] reported lower ALP in the Ti-6Al-4V, when compared to CpTi and Ti-Nb-Zr-Ta quaternary alloy, differing from our study. Regarding ALP molecular expression, experimental alloys exhibited increased expression, specially the Ti-13Nb-13Zr, which statistically differed from Ti-6Al-4V alloy; in agreement with Sista et al. (2013)[42], which also reported higher ALP expression in experimental alloys. We suggest that the highest production of ALP, from molecular expression data in Ti-13Nb-13Zr alloy, indicates that this material may accelerate cell differentiation, stimulating cells to synthesize ALP.

This hypothesis is in accordance with the result of OC expression; in which Ti-13Nb-13Zr and Ti-35Nb-7Zr-5Ta presented the highest values. We suggest that these results indicate more accelerated maturation of cells in these alloys, whereas OC is considered a late phase marker of osteoblast cell differentiation and matrix mineralization [43].

Matrix mineralization is the final event of bone formation, being considered a functional parameter in the *in vitro* studies that use osteogenic cells, since they reflect the progress of cell differentiation [44]. Guo et al., (2013)[4] reported that quaternary alloys promoted a greater formation of mineralized matrix, compared to CpTi, as observed in the present study, in nodule analysis. Analyzing the cellular assays of matrix nodules and the molecular expression of OP together, Ti-35Nb-3Zr-2Ta and Ti-35Nb alloys showed the highest values for both. Surprisingly, Ti-13Nb-13Zr alloy showed the lowest mean in these two analyzes.

The cascade of events behind implant may include the release of pro-inflammatory cytokines and the production of pro-resorptive cytokines [45]. A crucial role has been attributed to IL-6 and TNF-α cytokines, which are release in the presence of metal particles [46] and also due to the chemical composition of the material[47]. Despite its bone resorption ability, IL-6 can also act as an anti-inflammatory agent[48], due to its influence on osteoblast proliferation, differentiation and apoptosis[49]. Regarding TNF-α, Osta et al. (2014)[50] reported that it can also be active in the osteoblastogenese. In this present study, both cytokines were expressed in increased values for experimental alloys, specially IL-6, that statistically differed Ti-6Al-4V alloy. Ma et al. (2014)[51] also observed enhanced Il-6 values in nano structured titanium in comparison to control surface, and similar our result, the authors also verified better bone formation in material with higher expression of IL-6. Interestly the experimental alloys did not show significant difference when compared with CpTi, which is gold standard for biomaterial.

Contrary to the action of pro-inflammatory cytokines, transforming growth factor beta 1 (TGF-β1), acts on the activation of osteoblasts for bone matrix synthesis, promoting an earlier osseointegration [36]. TGF-β1 acts on osteoblast differentiation as a signal for the production of molecules, such as SMADs, that will act on transcription factors for osteoblastic differentiation, such as Runx2[52]. In this context, a positive relationship between the high production of TGF and Runx2 was observed in the experimental alloy Ti-35Nb-3Zr-2Ta, especially in the period of 14 days. However, when the expression of Runx2 was analyzed alone, as demonstrated previously by Sista et al., (2013)[42], there is no difference among the experimental alloys, CpTi and Ti-6Al-4V groups.

There are few previous research about experimental alloys with porous surface submitted to microtomography and histomorphometry analysis. The analysis performed on this study showed that in the period of 2 weeks, in general, the experimental alloys exhibited increased values, especially Ti-13Nb-13Zr and Ti-35Nb-3Zr-2Ta alloys, sometimes with statistically difference when compared to CpTi and Ti-6Al-4V alloys, dependent of parameter. Most values, at 8 weeks, did not present significant difference among experimental alloys and CpTi or Ti-6Al-4V in microtomography analysis but in the histomorphometric analysis, the experimental alloys presented more bone ingrowth than CpTi and Ti-6Al-4V. These positive results of experimental alloys were similar to recent previous studies that also observed better results for bone formation in experimental alloys [2, 6, 22, 31].

## Conclusion

The ternary and quaternary experimental Ti-13Nb-13Zr and Ti35Nb-7Zr-5Ta alloys presented positive results in the *in vitro* and *in vivo* analysis. Furthermore the characteristics (pore, porosity, geometric and elastic modulus) of these alloys were similar to that exhibited by the positive control TiCp. So, it seems reasonable to consider these alloys remarkable materials for development of dental implants, such as implant design presenting porous surface integrated with a dense core.

## Supporting information

S1 Table Results of column statistics (mean, standard deviation, standard error of mean and Kolmogorov Smirnov normality test to data from alkaline phosphatase assay. (PDF)

S2 Table Results of column statistics (mean, standard deviation, standard error of mean and Kolmogorov Smirnov normality test to data from total protein content assay. (PDF)

## References

[1] Dewidar MM, Yoon HC, Lim JK. Mechanical properties of metals for biomedical applications using powder metallurgy process: A review. Metals and Materials International. 2006;12:193–206.

[2] Bottino MC, Coelho PG, Yoshimoto M, König B Jr, Henriques VAR, Bressiani AHA, et al Histomorphologic evaluation of Ti-13Nb-13Zralloys processed via powder metallurgy. Materials Science and Engineering: C 2008;28:223–7.

[3] Niinomi M, Nakai M, Hieda J. Development of new metallic alloys for biomedical applications. Acta Biomater. 2012;8:3888–903. doi: 10.1016/j.actbio.2012.06.037 22765961 10.1016/j.actbio.2012.06.037

[4] Guo Y, Chen D, Cheng M, Lu W, Wang L, Zhang X. The bone tissue compatibility of a new Ti35Nb2Ta3Zr alloy with a low Young's modulus. Int J Mol Med. 2013;31:689–97. doi: 10.3892/ijmm.2013.1249 23338484 10.3892/ijmm.2013.1249

[5] Park YJ, Song YH, An JH, Song HJ, Anusavice KJ. Cytocompatibility of pure metals and experimental binary titanium alloys for implant materials. J Dent. 2013;41:1251–8. doi: 10.1016/j.jdent.2013.09.003 24060476 10.1016/j.jdent.2013.09.003

[6] Weng X, Yang H, Xu J, Li X, Liao Q, Wang J. In vivo testing of porous Ti-25Nb alloy serving as a femoral stem prosthesis in a rabbit model. Exp Ther Med. 2016;12:1323–30. doi: 10.3892/etm.2016.3472 27602063 10.3892/etm.2016.3472 PMC4998353

[7] Bottino MC, Coelho PG, Henriques VA, Higa OZ, Bressiani AH, Bressiani JC. Processing, characterization, and in vitro/in vivo evaluations of powder metallurgy processed Ti-13Nb-13Zr alloys. J Biomed Mater Res A. 2009;88:689–96. doi: 10.1002/jbm.a.31912 18335528 10.1002/jbm.a.31912

[8] Sista S, Wen C, Hodgson PD, Pande G. Expression of cell adhesion and differentiation related genes in MC3T3 osteoblasts plated on titanium alloys: role of surface properties. Mater Sci Eng C Mater Biol Appl. 2013;33:1573–82. doi: 10.1016/j.msec.2012.12.063 23827610 10.1016/j.msec.2012.12.063

[9] Niinomi M. Metallic biomaterials. J Artif Organs. 2008;11:105–10. doi: 10.1007/s10047-008-0422-7 18836869 10.1007/s10047-008-0422-7

[10] Khan MA, Williams RL, Williams DF. The corrosion behaviour of Ti-6Al-4V, Ti-6Al-7Nb and Ti-13Nb-13Zr in protein solutions. Biomaterials. 1999;20:631–7. 10208405 10.1016/s0142-9612(98)00217-8

[11] Andrade DP, Vasconcellos LM, Carvalho IC, Forte LF, Souza Santos EL, Prado RF, et al Titanium-35niobium alloy as a potential material for biomedical implants: In vitro study. Mater Sci Eng C Mater Biol Appl. 2015;56:538–44. doi: 10.1016/j.msec.2015.07.026 26249625 10.1016/j.msec.2015.07.026

[12] Prado RF, Rabelo SB, Andrade DP, Nascimento RD, Henriques VA, Carvalho YR, et al Porous titanium and Ti-35Nb alloy: effects on gene expression of osteoblastic cells derived from human alveolar bone. J Mater Sci Mater Med. 2015;26:259 doi: 10.1007/s10856-015-5594-0 26449449 10.1007/s10856-015-5594-0

[13] Park JW, Jang IS, Suh JY. Bone response to endosseous titanium implants surface-modified by blasting and chemical treatment: a histomorphometric study in the rabbit femur. J Biomed Mater Res B Appl Biomater. 2008;84:400–7. doi: 10.1002/jbm.b.30884 17595031 10.1002/jbm.b.30884

[14] Cordeiro JM, Beline T, Ribeiro ALR, Rangel EC, da Cruz NC, Landers R, et al Development of binary and ternary titanium alloys for dental implants. Dent Mater. 2017;33:1244–57. doi: 10.1016/j.dental.2017.07.013 28778495 10.1016/j.dental.2017.07.013

[15] Santos DR, Pereira MS, Cairo CAA, Graca MLA, Henriques VAR. Isochronal sintering of the blended elemental Ti-35Nb alloy. Materials Science and Engineering a-Structural Materials Properties Microstructure and Processing. 2008;472:193–7.

[16] Niinomi M. Fatigue performance and cyto-toxicity of low rigidity titanium alloy, Ti-29Nb-13Ta-4.6Zr. Biomaterials. 2003;24:2673–83. 12711513 10.1016/s0142-9612(03)00069-3

[17] Hagihara K, Nakano T, Maki H, Umakoshi Y, Niinomi M. Isotropic plasticity of beta-type Ti-29Nb-13Ta-4.6Zr alloy single crystals for the development of single crystalline beta-Ti implants. Sci Rep. 2016;6:29779 doi: 10.1038/srep29779 27417073 10.1038/srep29779 PMC4945923

[18] Niinomi M. Mechanical biocompatibilities of titanium alloys for biomedical applications. J Mech Behav Biomed Mater. 2008;1:30–42. doi: 10.1016/j.jmbbm.2007.07.001 19627769 10.1016/j.jmbbm.2007.07.001

[19] Vasconcellos LMR, Oliveira MV, Alencastro-Graca ML, Vasconcellos LGO, Carvalho YR, Alves CAC. Porous Titanium Scaffolds Produced by Powder Metallurgy for Biomedical Applications. Mat Res. 2008;11:275–80.

[20] Vasconcellos LMR, Oliveira FN, Leite DO, Vasconcellos LGO, Prado RF, Ramos CJ, et al Novel production method of porous surface Ti samples for biomedical application. J Mater Sci Mater Med. 2012;23:357–64. doi: 10.1007/s10856-011-4515-0 22183791 10.1007/s10856-011-4515-0

[21] Xu J, Weng X-J, Wang X, Huang J-Z, Zhang C, Muhammad H, et al Potential Use of Porous Titanium-Niobium Alloy in Orthopedic Implants: Preparation and Experimental Study of Its Biocompatibility In Vitro. Plos One. 2013;8:e79289 doi: 10.1371/journal.pone.0079289 24260188 10.1371/journal.pone.0079289 PMC3834032

[22] Galli S, Jimbo R, Naito Y, Berner S, Dard M, Wennerberg A. Chemically modified titanium-zirconium implants in comparison with commercially pure titanium controls stimulate the early molecular pathways of bone healing. Clin Oral Implants Res. 2017;28:1234–1240. doi: 10.1111/clr.12947 27531124 10.1111/clr.12947

[23] Brentel AS, Vasconcellos LMR, Oliveira MV, Graca MLA, Vasconcellos LGO, Cairo CAA, et al Histomorphometric analysis of pure titanium implants with porous surface versus rough surface. J Appl Oral Sci. 2006;14:213–8. doi: 10.1590/S1678-77572006000300013 19089076 10.1590/S1678-77572006000300013 PMC4327200

[24] Vasconcellos LMR, Leite DO, Nascimento FO, Vasconcellos LGO, Graca MLA, Carvalho YR, et al Porous titanium for biomedical applications: An experimental study on rabbits. Med Oral PatolOral Cirugia Bucal. 2010;15:E407–E12. 19767696

[25] Capek J, Vojtech D. Properties of porous magnesium prepared by powder metallurgy. Mater Sci Eng C Mater Biol Appl. 2013;33:564–9. doi: 10.1016/j.msec.2012.10.002 25428111 10.1016/j.msec.2012.10.002

[26] Bencharit S, Byrd WC, Altarawneh S, Hosseini B, Leong A, Reside G, et al Development and applications of porous tantalum trabecular metal-enhanced titanium dental implants. Clin Implant Dent Relat Res. 2014;16:817–26. doi: 10.1111/cid.12059 23527899 10.1111/cid.12059 PMC3708989

[27] Deporter D, Pharoah M, Yeh S, Todescan R, Atenafu EG. Performance of titanium alloy sintered porous-surfaced (SPS) implants supporting mandibular overdentures during a 20-year prospective study. Clin Oral Implants Res. 2014;25:e189–95. doi: 10.1111/clr.12043 23039057 10.1111/clr.12043

[28] Peng W, Xu L, You J, Fang L, Zhang Q. Selective laser melting of titanium alloy enables osseointegration of porous multi-rooted implants in a rabbit model. Biomed Eng Online. 2016;15:85 doi: 10.1186/s12938-016-0207-9 27439427 10.1186/s12938-016-0207-9 PMC4955147

[29] Oyonarte R, Pilliar RM, Deporter D, Woodside DG. Peri-implant bone response to orthodontic loading: Part 1. A histomorphometric study of the effects of implant surface design. Am J Orthod Dentofacial Orthop. 2005;128:173–81. doi: 10.1016/j.ajodo.2004.02.023 16102401 10.1016/j.ajodo.2004.02.023

[30] Van der Stok J, Van der Jagt OP, Amin SY, Haas MF, Waarsing JH, Jahr H, et al Selective laser melting-produced porous titanium scaffolds regenerate bone in critical size cortical bone defects. J Orthop Res. 2013;31:792–9. doi: 10.1002/jor.22293 23255164 10.1002/jor.22293

[31] Shi L, Wang L, Duan Y, Lei W, Wang Z, Li J, et al The improved biological performance of a novel low elastic modulus implant. PLoS One. 2013;8:e55015 doi: 10.1371/journal.pone.0055015 23437048 10.1371/journal.pone.0055015 PMC3578840

[32] Oliveira PT, Nanci A. Nanotexturing of titanium-based surfaces upregulates expression of bone sialoprotein and osteopontin by cultured osteogenic cells. Biomaterials. 2004;25:403–13. 14585688 10.1016/s0142-9612(03)00539-8

[33] Lowry OH, Rosebrough NJ, Farr AL, Randall RJ. Protein measurement with the folin phenol reagent. J BiolChem. 1951;193:265–75. 14907713

[34] Gregory CA, Gunn WG, Peister A, Prockop DJ. An Alizarin red-based assay of mineralization by adherent cells in culture: comparison with cetylpyridinium chloride extraction. AnalBiochem. 2004;329:77–84. 10.1016/j.ab.2004.02.002 15136169

[35] Rosa AL, Crippa GE, Oliveira PT, Taba M Jr., Lefebvre LP, Beloti MM. Human alveolar bone cell proliferation, expression of osteoblastic phenotype, and matrix mineralization on porous titanium produced by powder metallurgy. Clin Oral Implants Res. 2009;20:472–81. 19250245 10.1111/j.1600-0501.2008.01662.x

[36] Liu XH, Wu L, Ai HJ, Han Y, Hu Y. Cytocompatibility and early osseointegration of nanoTiO2-modified Ti-24 Nb-4 Zr-7.9 Sn surfaces. Mater Sci Eng C Mater Biol Appl. 2015;48:256–62. doi: 10.1016/j.msec.2014.12.011 25579921 10.1016/j.msec.2014.12.011

[37] Teixeira LN, Crippa GE, Lefebvre LP, Oliveira PT, Rosa AL, Beloti MM. The influence of pore size on osteoblast phenotype expression in cultures grown on porous titanium. Int J Oral Maxillofac Surg. 2012;41:1097–101. doi: 10.1016/j.ijom.2012.02.020 22487807 10.1016/j.ijom.2012.02.020

[38] Lindner M, Bergmann C, Telle R, Fischer H. Calcium phosphate scaffolds mimicking the gradient architecture of native long bones. J Biomed Mater Res A. 2014;102:3677–84. doi: 10.1002/jbm.a.35038 24307071 10.1002/jbm.a.35038

[39] Park CH, Lee CS, Kim YJ, Jang JH, Suh JY, Park JW. Improved pre-osteoblast response and mechanical compatibility of ultrafine-grained Ti-13Nb-13Zr alloy. Clin Oral Implants Res. 2011;22:735–42. doi: 10.1111/j.1600-0501.2010.02053.x 21121961 10.1111/j.1600-0501.2010.02053.x

[40] Samuel S, Nag S, Nasrazadani S, Ukirde V, El Bouanani M, Mohandas A, et al Corrosion resistance and in vitro response of laser-deposited Ti-Nb-Zr-Ta alloys for orthopedic implant applications. J Biomed Mater Res A. 2010;94:1251–6. doi: 10.1002/jbm.a.32782 20694992 10.1002/jbm.a.32782

[41] Chen XB, Li YC, Du Plessis J, Hodgson PD, Wen C. Influence of calcium ion deposition on apatite-inducing ability of porous titanium for biomedical applications. Acta Biomater. 2009;5:1808–20. doi: 10.1016/j.actbio.2009.01.015 19223253 10.1016/j.actbio.2009.01.015

[42] Sista S, Wen C, Hodgson PD, Pande G. Expression of cell adhesion and differentiation related genes in MC3T3 osteoblasts plated on titanium alloys: role of surface properties. Mater Sci Eng Mater Biol Appl. 2013;33:1573–82. 10.1016/j.msec.2012.12.063 23827610

[43] Mizuno M, Fujisawa R, Kuboki Y. Type I collagen-induced osteoblastic differentiation of bone-marrow cells mediated by collagen-alpha2beta1 integrin interaction. J Cell Physiol. 2000;184:207–13. doi: 10.1002/1097-4652(200008)184:2<207::AID-JCP8>3.0.CO;2-U 10867645 10.1002/1097-4652(200008)184:2<207::AID-JCP8>3.0.CO;2-U

[44] Hoemann CD, El-Gabalawy H, McKee MD. In vitro osteogenesis assays: Influence of the primary cell source on alkaline phosphatase activity and mineralization. Pathologie Biologie. 2009;57:318–23. doi: 10.1016/j.patbio.2008.06.004 18842361 10.1016/j.patbio.2008.06.004

[45] Okafor CC, Haleem-Smith H, Laqueriere P, Manner PA, Tuan RS. Particulate endocytosis mediates biological responses of human mesenchymal stem cells to titanium wear debris. J Orthop Res. 2006;24:461–73. doi: 10.1002/jor.20075 16450379 10.1002/jor.20075

[46] Borish LC, Steinke JW. 2. Cytokines and chemokines. J Allergy Clin Immunol. 2003;111:S460–75. 12592293 10.1067/mai.2003.108

[47] Granchi D, Ciapetti G, Stea S, Savarino L, Filippini F, Sudanese A, et al Cytokine release in mononuclear cells of patients with Co-Cr hip prosthesis. Biomaterials. 1999;20:1079–86. 10382823 10.1016/s0142-9612(99)00004-6

[48] Catelas I, Campbell PA, Dorey F, Frausto A, Mills BG, Amstutz HC. Semi-quantitative analysis of cytokines in MM THR tissues and their relationship to metal particles. Biomaterials. 2003;24:4785–97. 14530076 10.1016/s0142-9612(03)00378-8

[49] Franchimont N, Wertz S, Malaise M. Interleukin-6: An osteotropic factor influencing bone formation? Bone. 2005;37:601–6. doi: 10.1016/j.bone.2005.06.002 16112634 10.1016/j.bone.2005.06.002

[50] Osta B, Benedetti G, Miossec P. Classical and Paradoxical Effects of TNF-alpha on Bone Homeostasis. Front Immunol. 2014;5:1–9. doi: 10.3389/fimmu.2014.00001 24592264 10.3389/fimmu.2014.00048 PMC3923157

[51] Ma QL, Zhao LZ, Liu RR, Jin BQ, Song W, Wang Y, et al Improved implant osseointegration of a nanostructured titanium surface via mediation of macrophage polarization. Biomaterials. 2014;35:9853–67. doi: 10.1016/j.biomaterials.2014.08.025 25201737 10.1016/j.biomaterials.2014.08.025

[52] Wu M, Chen G, Li YP. TGF-beta and BMP signaling in osteoblast, skeletal development, and bone formation, homeostasis and disease. Bone Res. 2016;4:16009 doi: 10.1038/boneres.2016.9 27563484 10.1038/boneres.2016.9 PMC4985055

