# Supplementary material for: In vitro and in vivo biological performance of porous Ti alloys prepared by powder metallurgy
Source: PLoS One. 2018 May 17;13(5):e0196169. doi: 10.1371/journal.pone.0196169 (PMC5957353; doi:10.1371/journal.pone.0196169)
Supplement: S1 Table — (PDF) [file pone.0196169.s001.pdf]

Col. stats		A	B	C	D	E	F	G	H	I	J	K	L
		TiCp (3 days)	i-6Al-4V (3 days	i-13Nb-13Zr (3 days	Ti-35Nb (3 days	-35Nb-7Zr -5Ta (3 days	egative Control (3 day	TiCp (10 days	i-6Al-4V (10 days	i-13Nb-13Zr (10 days	Ti-35Nb (10 days	-35Nb-7Zr -5Ta (10 days	egative Control (10 day
		Y	Y	Y	Y	Y	Y	Y	Y	Y	Y	Y	Y
1	Number of values	12	12	12	12	12	12	12	12	12	12	12	12
2													
3													
4	Mean	1.031	1.281	0.9359	0.9049	0.9979	1.043	0.3994	0.7698	0.7361	0.4685	0.5451	0.5816
5	Std. Deviation	0.2058	0.1713	0.2791	0.2452	0.2285	0.2322	0.2074	0.4981	0.4099	0.2853	0.3489	0.4796
6	Std. Error of Mean	0.05941	0.04944	0.08057	0.07079	0.06596	0.06702	0.05988	0.1438	0.1183	0.08235	0.1007	0.1385
7													
8	KS normality test												
9	KS distance	0.2362	0.2100	0.1478	0.1709	0.1531	0.3002	0.1810	0.2970	0.2208	0.2372	0.2885	0.2229
10	P value	0.0631	0.1570	0.2000	0.2000	0.2000	0.0038	0.2000	0.0044	0.1095	0.0607	0.0067	0.1020
11	Passed normality test (alpha=0.05)?	Yes	Yes	Yes	Yes	Yes	No	Yes	No	Yes	Yes	No	Yes
12	P value summary	ns	ns	ns	ns	ns	**	ns	**	ns	ns	**	ns
