# Supplementary material for: In vitro and in vivo biological performance of porous Ti alloys prepared by powder metallurgy
Source: PLoS One. 2018 May 17;13(5):e0196169. doi: 10.1371/journal.pone.0196169 (PMC5957353; doi:10.1371/journal.pone.0196169)
Supplement: S2 Table — (PDF) [file pone.0196169.s002.pdf]

Col. stats		A	B	C	D	E	F	G	H	I	J	K	L
		TiCp (3 days)	Ti-6Al-4V (3 days)	Ti-13Nb-13Zr (3 days)	Ti-35Nb (3 days)	Ti-35Nb-7Zr -5Ta (3 days)	Negative Control (3 days	TiCp (10 days)	Ti-6Al-4V (10 days)	Ti-13Nb-13Zr (10 days	Ti-35Nb (10 days)	Ti-35Nb-7Zr -5Ta (10 days	Negative Control (10 days
		Y	Y	Y	Y	Y	Y	Y	Y	Y	Y	Y	Y
1	Number of values	12	12	12	12	12	12	12	12	12	12	12	12
2													
3													
4	Mean	64.80	67.07	34.55	63.91	84.50	16.04	157.2	127.0	160.9	196.5	209.2	94.16
5	Std. Deviation	50.71	19.25	12.06	38.92	51.68	15.16	40.65	29.13	43.01	48.10	37.34	65.20
6	Std. Error of Mean	14.64	5.558	3.481	11.24	14.92	4.376	11.74	8.409	12.42	13.88	10.78	18.82
7													
8	Lower 95% CI of mean	32.59	54.83	26.89	39.18	51.66	6.409	131.4	108.5	133.6	166.0	185.4	52.74
9	Upper 95% CI of mean	97.02	79.30	42.21	88.63	117.3	25.67	183.1	145.5	188.3	227.1	232.9	135.6
10													
11	KS normality test												
12	KS distance	0.2226	0.1734	0.1905	0.1342	0.1363	0.3868	0.1479	0.2123	0.2127	0.2828	0.1683	0.3010
13	P value	0.1032	0.2000	0.2000	0.2000	0.2000	< 0.0001	0.2000	0.1454	0.1439	0.0088	0.2000	0.0036
14	Passed normality test (alpha=0.05	Yes	Yes	Yes	Yes	Yes	No	Yes	Yes	Yes	No	Yes	No
15	P value summary	ns	ns	ns	ns	ns	****	ns	ns	ns	**	ns	**
